# Enhancing Soil Water-Soluble Carbon Stability Structure Through Straw Return in Maize–Soybean Rotation in Mollisols

**DOI:** 10.3390/plants15101553

**Published:** 2026-05-19

**Authors:** Enjun Kuang, Lin Liu, Zixuan Wang, Jiuming Zhang, Yingxue Zhu, Di Zhu, Gilles Colinet, Baofeng Guo, Lei Sun

**Affiliations:** 1 Heilongjiang Academy of Black Soil Conservation and Utilization, Key Laboratory of Soil Environment and Plant Nutrition of Heilongjiang Province, Harbin 150086, China; kuangenjun2002@163.com (E.K.);; 2 College of Agriculture, Heilongjiang Bayi Agricultural University, Daqing 163319, China; 3 Department of Biosystems Engineering (BIOSE), Gembloux Agro-Bio Tech of Université de Liège, 5030 Gembloux, Belgium; 4 Central Reserve Grain Nenjiang Direct Warehouse Co., Ltd., 93 Branch, Nenjiang 161441, China

**Keywords:** maize–soybean rotation, straw return, water-soluble organic carbon, three-dimensional fluorescence spectra, PARAFAC analysis

## Abstract

This study investigated the effects of different straw return practices—no-tillage with straw mulching (SM), shallow tillage with straw incorporation (SS), and deep tillage with straw incorporation (DS)—on the content and structural characteristics of soil water-soluble organic carbon (WSOC) under a maize–soybean rotation in the black soil region in the Northeast of China. Compared with SM, SS and DS increased WSOC content by 39.0% and 28.8% in the 0~20 cm layer (*p* < 0.05), and by 28.4% and 8.5% in the 20–40 cm layer, respectively. Deep tillage combined with straw return reduced the WSOC/SOC ratio. The DS treatment exhibited the highest levels under maize straw incorporation, while SM treatment showed the highest levels under soybean straw incorporation. Spectral indices in both maize and soybean seasons—including the fluorescence index (FI, ranging from 1.53 to 1.57 in the maize season and from 1.53 to 1.67 in the soybean season), biological index (BIX, ranging from 0.84 to 1.79 in the maize season and from 0.61 to 0.74 in the soybean season), and humification index (HIX, ranging from 0.51 to 0.79 in the maize season and from 0.84 to 0.97 in the soybean season)—collectively indicated that WSOC predominantly consisted of microbially processed organic matter with a low degree of humification. PARAFAC modeling resolved two fluorescent components in maize season: C1 (humic acid-like substances, accounting for 34.8–54.9%) and C2 (Tryptophan-like substance, accounting for 45.1–65.2%), and two components in the soybean season: C1 (humic-like substances, 51.0–53.7%), and C2 (Fulvic acid-like substance 46.3–49.0%). Overall, deep straw return promotes soil humification but increases the structural complexity of WSOC. This systematic investigation provides mechanistic insights into how straw return practices regulate the quantity and quality of labile carbon pools in agricultural ecosystems over time.

## 1. Introduction

Water-soluble organic carbon (WSOC) is a key component of soil soluble organic carbon and plays an important role in the active carbon and nutrient pools of agricultural soils. It serves as a direct source of organic carbon for microbial uptake and utilization [1]. The biodegradable portion of WSOC typically accounts for 10–40% of the total [2]. WSOC is highly sensitive to various factors such as land-use patterns, fertilization practices, crop species, and microbial activity, maintaining a dynamic equilibrium. Consequently, it is widely regarded as a sensitive indicator of the changes in soil organic carbon (SOC) [3,4,5]. In agricultural production, the return of organic materials to the soil represents the main pathway for exogenous organic carbon input, thus directly influencing the content and composition of WSOC. These effects are further modulated by tillage practices and the frequency of straw incorporation [5], which together can lead to varying degrees of WSOC humification [6,7]. Moreover, combining straw incorporation with deep tillage can enhance the organic carbon pool in soil layers up to 40 cm, thereby improving nutrient availability in the subsoil [8]. Traditionally, the sources and composition of soil dissolved organic matter (DOM) have been characterized using three-dimensional excitation–emission matrix fluorescence spectroscopy coupled with parallel factor analysis (PARAFAC) [9,10,11]. This approach enables the resolution of DOM components, provides insights into the degree of humification and soil organic matter activity [12], and has been widely applied in agricultural and environmental ecosystems. Previous studies indicate that straw incorporation increases the proportion of fulvic acid within the fluorescent fraction of organic carbon, accompanied by simplification of its molecular structure [13].

Long-term continuous cropping has been shown to reduce soybean yield, deteriorate quality, impair growth and development, and increase pest and disease incidence [14,15]. Liu [16] investigated the conservation status of black soils in Northeast China and concluded that implementing rational crop rotation systems can improve black soil quality and mitigate the adverse effects of continuous cropping of soybean and maize. Under maize–soybean rotation, the yields of maize and soybean increased by 1.0 and 0.4 t/ha, respectively [17]. Promoting this rotational system is therefore a key strategy for improving the soil microenvironment, enhancing the plow layer structure, and supporting high grain yields [18,19]. As a dominate cropping system in Heilongjiang Province, Northeastern China, the maize–soybean rotation leverages the advantage of the biological nitrogen fixation of soybean nodules to supply nitrogen for the subsequent maize crop. In return, the soil conditions fostered by maize cultivation benefit biological nitrogen fixation in soybean [20], thereby establishing a mutually beneficial cycle. This rotation system also alleviates the problems associated with continuous monocropping, enhances soil fertility, and aids in the control pests, weeds, and diseases, ultimately contributing to increased crop productivity [21,22]. Moreover, crop-specific straw incorporation methods, where maize and soybean residues are returned to distinct soil depths, enables full-profile fertility improvement across the plow layer [23]. This approach effectively addresses challenges such as the large volume of straw and its slow decomposition following repeated maize straw return.

We hypothesized that the maize straw incorporation influences the fluorescent fractions of WSOC in soil. Accordingly, the main objectives of this study were to: (1) determine whether crop residue return, via fallow management and deep straw incorporation, increases WSOC content, and (2) investigate whether maize straw incorporation introduces more stable organic substances during the soybean growing season. This study systematically analyzed the effects of different straw return practices on WSOC content and structural characteristics under maize–soybean rotation in Mollisols, thereby providing a basis for enhancing carbon sequestration and soil fertility in rotational cropping systems.

## 2. Materials and Methods

### 2.1. Site Description

The study was conducted at the experimental station of the Heilongjiang Academy of Agricultural Sciences (45°58′–46°10′ N, 132°46′–133°15′ E), located in Minzhu Township, Heilongjiang Province, China. The soil was a typical black soil classified as Mollisols, according to IUSS Working Group WRB [24]. This area features a temperate continental monsoon climate with a mean annual temperature of 3.5 °C, an annual accumulated temperature (≥10 °C) of 2442.8 °C, and a frost-free period of 131 days. Precipitation is concentrated from June to September. A maize–soybean rotation system has a single-cropping regimen per year. The basic soil properties before the establishment of the experiment were: organic matter 26.1 g·kg^−1^, alkali-hydrolysable nitrogen 101.2 mg·kg^−1^, available phosphorus 26.3 mg·kg^−1^, available potassium 200.0 mg·kg^−1^, and pH 6.6.

### 2.2. Experimental Design

This trial was established in the autumn of 2021 with a maize–soybean rotation system. Three treatments were implemented: deep straw incorporation (DS), shallow straw incorporation (SS), and straw mulching (SM). Each treatment was replicated three times in a randomly arranged plot with an area of 65 m^2^. Agronomic practices were employed equally across all the plots, such as fertilization, irrigation, and pest control. During the mechanical harvesting of maize in autumn, the straw was simultaneously chopped into a length of ≤12 cm and mulched on the ground using a John Deere S760 combine harvest (339HP) with an integrated shredding system. For the deep and shallow straw returning treatments, a Leveo Leopard M2104-6 (G4) Four-Wheel Tractor was used to plow and bury the straw together with the soil to the depths of 0–15 cm and 0–35 cm. The same method was used in the soybean season. Full straw return was applied, with approximately 15,000 kg·ha^−1^ returned in the maize season and about 4800 kg·ha^−1^ in the soybean season, where soybean straw was chopped to ≤10 cm.

In spring, ridges were manually formed, followed by fertilization and sowing. Maize (cultivated variety Demia No.2) was planted in 2022 with fertilizer application rates of 150 kg N ha^−1^, 75 kg P_2_O_5_ ha^−1^, and 75 kg K_2_O ha^−1^. Soybean (cultivated variety Heinong 48) was sown in 2023 with fertilizer rates of 50 kg N ha^−1^, 55 kg P_2_O_5_ ha^−1^, and 45 kg K_2_O ha^−1^. Uniform field managements were applied across all the treatments. No irrigation was conducted during the plant growth stage.

Soil samples were collected in autumn 2022 and 2023. For each treatment, soil was collected from the 0–20 cm and 20–40 cm layers using an S-shaped sampling pattern. The collected samples were thoroughly mixed, and visible crop residues and small stones were removed. The desiccated samples were sequentially sieved through 2 mm and 0.01 mm mesh screens, using mechanical shakers for spectroscopic and chemical analyses.

### 2.3. Measurement Methods

SOC measurement: Air-dried soil samples were used to determine SOC. Approximately 0.0100 g of soil was accurately weighed and digested with 2 mol·L^−1^ HCl. The digest was filtered through a 0.45 µm membrane, and the SOC content was measured using a total organic carbon analyzer (multi-N/C 3100, Analytik Jena, Germany) [25].

WSOC measurement: For WSOC, 3 g of air-dried soil was mixed with 30 mL of ultrapure water. The suspension was shaken horizontally at 200 r·min^−1^ for 24 h at room temperature. After centrifugation at 12,000 r·min^−1^ for 20 min, the supernatant was filtered through a 0.45 μm membrane and then analyzed for WSOC concentration using the same TOC analyzer [26].

Fluorescence EEM: To minimize the concentration effects on the fluorescence measurements, an aliquot of the filtered supernatant from each treatment was diluted with ultrapure water to a uniform WSOC concentration of 10 mg·L^−1^. EEM fluorescence spectra were recorded on a Hitachi F-7000 fluorescence spectrometer (Tokyo, Japan) at excitation (Ex) and emission (Em) wavelengths from 200 to 600 nm, with a 10 nm bandwidth and a scan speed of 1200 nm·min^−1^. Ultrapure water served as the blank [27].

Fluorescence index (FI): FI was calculated as the ratio of emission intensity at 450 nm to that at 500 nm under excitation at 370 nm [28]. FI values < 1.4 indicate that WSOC originates mainly from external plant materials (e.g., litter and root exudates); values > 1.9 point to microbial sources (soil metabolism and degradation); and values between 1.4 and 1.9 suggest mixed contributions [29].

Biological index (BIX): BIX was calculated as the emission ratio at 380 nm to 430 nm with excitation at 310 nm [30]. BIX values of 0.6–0.7 reflect low autochthonous contribution; 0.7–0.8 indicate moderate recently produced autochthonous components; 0.8–1.0 imply a strong contribution from recently formed autochthonous WSOC; and values > 1.0 signify predominantly autochthonous and newly generated organic matter [29].

Humification index (HIX): HIX was determined as the ratio of integrated emission intensity over 435–480 nm to the sum of integrated intensities over 435–480 nm and 300–345 nm (excitation at 254 nm). Higher HIX values indicate a greater degree of humification [31].

PARAFAC analysis: PARAFAC was performed in MATLAB using the DOMFlour toolbox [32]. Before modeling, Raman scattering effects were removed by subtracting the blank signal, followed by interpolation across the Raman scattering regions.

### 2.4. Statistical Analysis

Statistical analysis was performed using Excel 2010 and SPSS 22.0. One-way analysis of variance (ANOVA) followed by Duncan’s multiple range test (α = 0.05) was applied for multiple comparisons. Pearson correlation analysis of soil SOC, WSOC, and related component indicators was conducted using Origin 2021. The three-dimensional fluorescence spectra were plotted, and parallel factor analysis (PARAFAC) was performed using MATLAB 2020. Fluorescence spectral indices were calculated through area integration using Origin 2021. Data in the figures and tables are presented as mean ± standard deviation.

## 3. Results

### 3.1. Dynamics of SOC in Different Treatments

**Figure 1:**
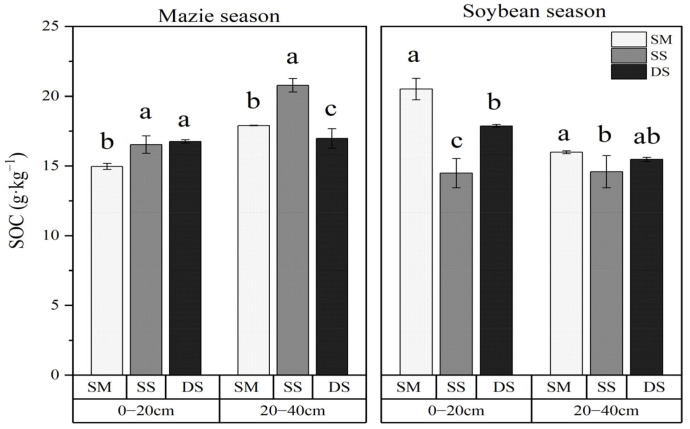
SOC under different treatments in 0–40 cm soil profiles. Lowercase letters denote significant differences (Duncan’s multiple range test, *p* < 0.05) among treatments within the same soil layer. Error bars represent standard deviations (*n* = 3). SM: straw mulching; SS: shallow straw incorporation; and DS: deep straw incorporation.

The SOC content under different treatments is presented in Figure 1. During maize season, straw incorporation at varying depths significantly increased SOC concentrations in both the surface (0–20 cm) and subsurface (20–40 cm) layers compared to the SM control (*p* < 0.05). Specifically, at the 0–20 cm soil layer, the SS and DS treatments enhanced the SOC content by 10.4% and 12.0% (*p* < 0.05); however, at 20–40 cm depth, SOC content was lowest under DS treatment and highest under SS, with SS showing a 16.1% increase over SM treatment (*p* < 0.05).

In contrast, during the soybean season, SOC content under SM treatment was significantly higher than under both SS and DS treatments at both soil depths (*p* < 0.05). At 0–20 cm, SS and DS treatments reduced SOC by 29.4% and 12.9%, respectively, compared to SM; at 20–40 cm, the reduction was 8.7% and 3.2% (*p* < 0.05).

### 3.2. Dynamics of WSOC and WSOC/SOC Ratios

**Figure 2:**
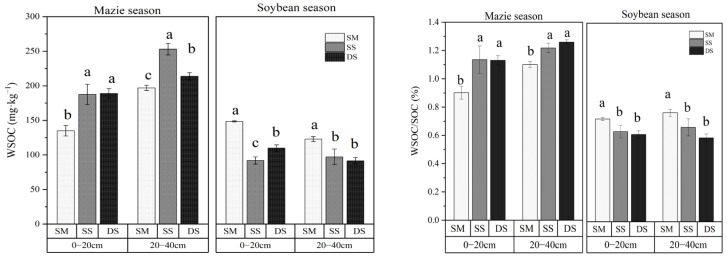
WSOC content and WSOC/SOC ratios under different treatments in 0–40 cm soil profiles. Lowercase letters denote significant differences (Duncan’s multiple range test, *p* < 0.05) among treatments within the same soil layer. Error bars represent standard deviations (*n* = 3). SM: straw mulching; SS: shallow straw incorporation; and DS: deep straw incorporation.

As demonstrated in Figure 2, WSOC content exhibited that deep straw return increased in maize season and had a progressive decline in soybean season with increasing soil depth across all treatments. Straw incorporation significantly enhanced WSOC concentrations in both the surface (0–20 cm) and subsurface (20–40 cm) layers compared to the SM control (*p* < 0.05). In maize season, compared with SM treatment, SS and DS treatments increased the WSOC content with increases of 39.0% and 28.8% at the 0–20 cm soil layer (*p* < 0.05), and at the 20–40 cm soil layer, the WSOC content was higher than the SM treatment by 28.4% and 8.5%, respectively (*p* < 0.05). But, in soybean season, the WSOC content of the SM treatment had a significantly higher difference than the SS and DS treatments both in the 0–20 cm and 20–40 cm soil layers (*p* < 0.05), while the content of WSOC in the SM treatment had a significantly higher difference than SS and DS by 61.6% and 35.2% at the 0–20 cm soil layer, while they were 26.4% and 34.4% higher in the 20–40 cm (*p* < 0.05).

The amount of maize straw returned to different soil layers increased the WSOC content of the 0–40 cm soil layer. Conversely, in the soybean season, little soybeans on the surface soil increased the WSOC content significantly; when the shallow plow and deep plow were scattering the soybean straw, this had no significant effect on the increased WSOC content.

The WSOC/SOC ratio is widely used as an indicator of SOC lability and responsiveness to agricultural management practices. A higher ratio reflects greater SOC activity. In the 0–20 cm layer, the WSOC/SOC ratio of the SS treatment was higher than SM and DS of 25.9% and 35.5%, and SS and DS were 10.6% and 14.4% higher than the SM treatment at 20–40 cm in the maize season. In the soybean season, the SM treatment had a higher WSOC/SOC ratio than SS and DS both in the 0–20 cm and 20–40 cm soil layer, by 14.1% and 17.8%, and 15.5% and 30.2%, respectively. Overall, deep tillage with straw incorporation increased the activity of WSOC/SOC in the maize season, and the covered straw had a higher ratio in the soybean season.

### 3.3. PARAFAC-Derived Fluorescence Components

**Figure 3:**
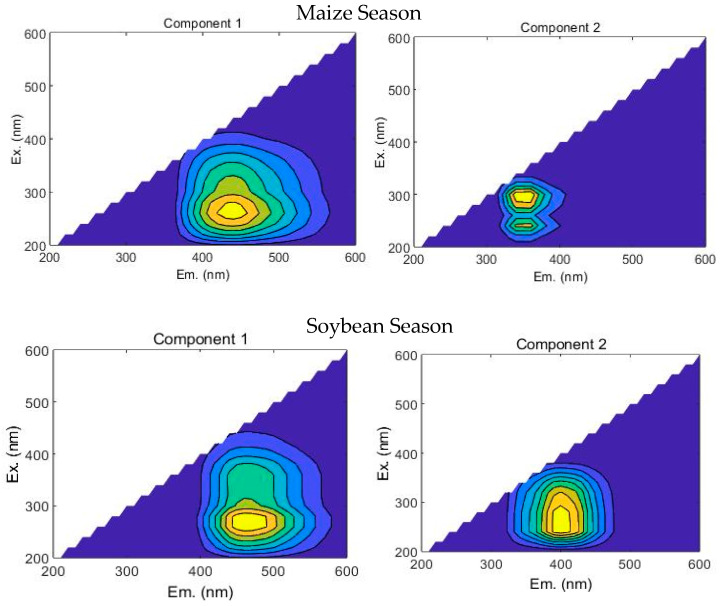
Fluorescence components of WSOC, based on the PARAFAC analysis method in the maize and soybean seasons. Ex: excitation wavelength and Em: emission wavelength. Maize season: C1—humic-like substances and C2—tryptophan-like substance. Soybean season: C1—humic-like substances and C2—fulvic acid-like substance.

After parallel factor (PARAFAC) analysis and the split-half of the excitation and emission matrix, two EEM-PARAFAC components were extracted during the maize and soybean growing seasons (Figure 3). The two components, C1 and C2, explained the 99.8% variability of the samples.

In the maize season:

C1: Humic-like substances (Ex/Em: 260 nm/430–450 nm), characterized by a single excitation-emission peak in the ultraviolet region.

C2: Tryptophan-like substance (Ex/Em: 300 nm/340~360 nm), displaying dual excitation maxima associated with carboxyl-rich proteins.

In the soybean season:

C1: Humic-like substances (Ex/Em: 260 nm/460–470 nm), characterized by a single excitation-emission peak in the ultraviolet region. It is a highly humified and structurally complex terrestrial humus, with a high degree of aromatic condensation and molecular weight, representing a stable and slow-moving carbon pool in soil [33].

C2: Fulvic acid-like substance (Ex/Em: 240~280 nm/400 nm), displaying a single excitation-emission peak in the visible light region. Represents humic substances with low molecular weight and moderate humification degree, indicating their contribution as microbial sources in soil [33].

### 3.4. Proportion of Three-Dimensional Fluorescence Components in WSOC Under Different Treatments

**Figure 4:**
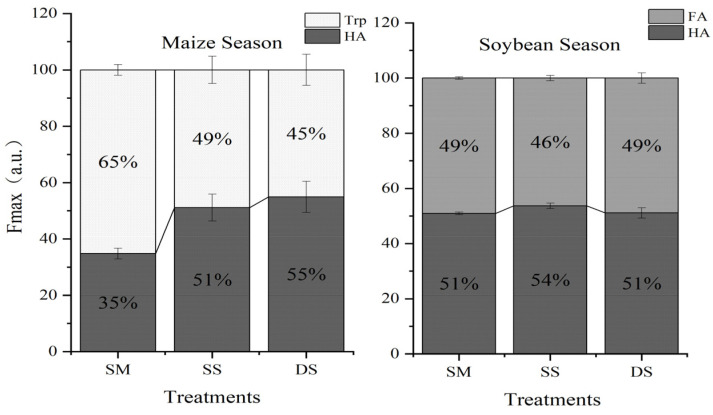
Fluorescence intensity and relative percentage of soil WSOC fluorescence components. Note: Different superscripts within columns indicate significant differences (Duncan’s test, *p* < 0.05). *n* = 3. HA: humic acid-like substances; Trp: Tryptophan-like substance; FA: Fulvic acid-like substance; SM: straw mulching; SS: shallow straw incorporation; and DS: deep straw incorporation.

Comparison of maximum fluorescence (Fmax) values revealed that humic-like substances (C1) dominated the fluorescent pool (34.8–54.9%), followed by a tryptophan-like substance (C2, 45.1%~65.2%) in maize season, while humic-like substances (C1) dominated the fluorescent pool (51.0–53.7%), followed by fulvic acid-like substance (C2) dominated the fluorescent pool (46.3–49.0%) in soybean season, shown in Figure 4.

The intensity of fluorescent substance components varies at different depths of straw returning to the soil during the maize season. As the depth of straw return increases, SM, SS, and DS type fulvic acid substances show an increasing trend, with the proportions of components in the three treatments being 34.8%, 51.1%, and 54.9%, respectively. On the contrary, the proportion of tryptophan-like substances showed a decreasing trend, at 65.2%, 48.9%, and 45.1%, respectively.

During the soybean season, two substances were also identified through PARAFAC, but they were humic-like substances and fulvic acid-like substances. As the depth of straw returning increased, the humic-like substances first increased and then decreased, with component proportions of 51.0%, 53.7%, and 51.1%, respectively. On the contrary, the proportion of fulvic acid-like substances increased with the depth of straw returning to the field, reaching 49.0%, 46.3%, and 49.9%, respectively.

### 3.5. Fluorescence Spectral Characteristics of WSOC in Soil Under Different Treatments

The fluorescence indices revealed distinct compositional differences among treatments, as shown in Table 1. The fluorescence index (FI), which typically reflected the source of humic substances, ranged from 1.53 to 1.57 during the maize season, with an average value of 1.54. In the soybean season, it ranged from 1.53 to 1.67, with an average value of 1.58. The values suggested that all treatments were affected by a combination of exogenous and endogenous substances. The biological index (BIX), which measured the contribution of endogenous organic matter, ranged from 0.84 to 1.79, with an average of 1.12 during the maize season. In the soybean season, it was considerably lower, ranging from 0.61 to 0.74 (average of 0.67). After maize straw incorporation, BIX values were significantly higher than those in the soybean season, with the latter decreasing by 40.2%. The higher BIX during the maize season indicated stronger autochthonous characteristics, a higher degree of humification, and a greater tendency to produce autochthonous carbon-derived products. Furthermore, the production of these autochthonous carbon-derived substances was higher in the surface soil than in the deeper soil.

The humification index (HIX), which represented the degree of humification of soil organic matter, also varied between the two crop seasons. Following maize straw incorporation, HIX values ranged from 0.51 to 0.79, with an average of 0.7. In contrast, values were notably higher during the soybean season, ranging from 0.84 to 0.91 and averaging 0.87. This represented a 24.3% increase in the average HIX for the soybean season compared to the maize season. Regarding the straw application methods, the humification coefficient was lowest in the 0–20 cm soil layer, where straw was applied on the surface. No significant differences in the humification coefficient were observed among the other treatments.

### 3.6. Correlation Coefficients Between WSOC Content, Fluorescence Components, and Parameters with Different Treatments

**Figure 5:**
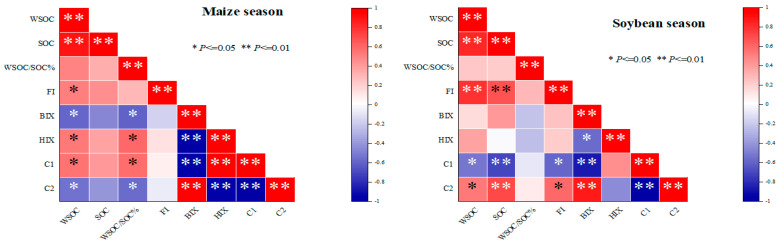
Correlation coefficients between soil WSOC and each fluorescence index. WSOC: water-soluble organic carbon; SOC: soil organic carbon; FI: fluorescence index; BIX: biological index; and HIX: humification index. C1 and C2: Different fluorescence spectral components by PARAFAC.

The correlation coefficients between WSOC content and other fluorescence components and parameters during the maize and soybean growing seasons are shown in Figure 5. In the maize season, WSOC exhibited a highly significant positive correlation with SOC, as well as a significant positive correlation with FI, HIX, and component C1. In contrast, WSOC showed a significant negative correlation with BIX and component C2. Additionally, BIX, HIX, C1, and C2 were strongly correlated with each other, showing both positive and negative relationships. SOC was not correlated with other indicators except for a positive correlation with WSOC.

In the soybean season, WSOC again displayed a highly significant positive correlation with SOC and FI. Component C2 showed significant positive correlation with SOC, WSOC, FI, and BIX, while it was negatively correlated with C1. Conversely, C1 exhibited the opposite trends. Furthermore, SOC was significantly positively correlated with FI and C2 and significantly negatively correlated with C1 during the soybean season.

## 4. Discussion

### 4.1. Influence of Different Treatments on the SOC and WSOC

Long-term continuous cultivation of farmland soil gradually depletes soil nutrients. However, straw incorporation combined with appropriate tillage practices can increase the SOC content, exerting a significant positive effect on the soil quality [34]. Straw return also activates soil nitrogen, phosphorus, and potassium nutrients, promotes their accumulation [35], and enhances the activity of beneficial microorganisms, thereby improving the soil microenvironment [36].

Different straw return practices increased the soil organic matter content in both the topsoil and subsoil. No-tillage with straw mulching enhanced topsoil organic carbon content [37], promoting the production and accumulation of labile soil organic matter. Deep straw incorporation increased the organic matter content in both the topsoil and subsoil and has been reported to simplify the structure of humic substances [38]. In this study, deep straw incorporation was more effective than surface straw mulching in increasing SOC and WSOC during the maize straw season. Returning straw to deeper soil layers also significantly increased subsoil organic carbon, consistent with findings on chernozem and black soil [38]. In contrast, during the soybean growing season, straw mulching led to higher WSOC content in the 0–40 cm soil layer than other treatments. It might be attributed to the low lignin content of soybean straw, which facilitated rapid decomposition and mineralization. Under surface mulching, soybean straw remained relatively concentrated, whereas in the shallow and deep straw treatments—where the straw was incorporated into the soil by tillage—a smaller amount of straw was mixed with the soil. Therefore, longer-term experiments are needed to validate these findings.

The WSOC/SOC ratio is commonly used as an indicator of organic carbon lability. A higher ratio reflects greater organic carbon lability, increased susceptibility to microbial decomposition, and higher sensitivity to management practices [39]. Straw return exhibited higher WSOC/SOC ratios, indicating reduced stability of the SOC pool [40,41], and greater biodegradability. In this study, straw mulching resulted in a lower WSOC/SOC ratio than deep straw return, suggesting that the latter had relatively low SOC stability [42]. Conversely, the value of the WSOC/SOC ratio of straw mulching was higher than deep straw incorporation treatments, which was also, due to the aforementioned reasons, contributing to enhanced stability of SOC. Deep straw returning enhanced the stability of SOC.

### 4.2. Influence of Different Treatments on WSOC Component

Incorporating maize straw into the soil provides a substantial carbon source for microbial utilization. In northeast China, the rowing season is limited to May–September, followed by a prolonged freeze–thaw period with low temperatures. Consequently, maize straw cannot be fully decomposed within a single growing season. The decomposition rate of deeply buried maize straw is only 60–70% in the first year in this region [43], leaving recalcitrant materials such as lignin residual in the soil. In this study, only residual maize straw from the previous season was considered because of the limited input of soybean straw. Structural analysis of WSOC revealed two distinct components in both the maize and soybean growing seasons, respectively. During the maize growing season, these components were humic acid-like (accounting for 34.8–54.9%) and protein-like compounds (accounting for as much as 45.1–65.2%). This distribution reflects the short-term plant–microbe interactions, following the return of large quantities of fresh maize straw. The incorporation of fresh straw introduces abundant carbon and nutrients into the soil, stimulates microbial activity, and increases the proportion of labile compounds. This stage also represents the initial stage of humification of fresh organic materials, characterized by the gradual transformation from simple, low-molecular-weight substances into more complex and stable organic compounds.

In the soybean growing season, two WSOC components were identified: fulvic acid-like (of 51.0–53.7%) and humic acid-like substances (of 46.3–49.0%). The proportion of humic acid-like substances gradually increased, whereas protein-like substances progressively converted into fulvic acid-like materials, which are intermediate products with a moderate degree of humification. This pattern indicated a process of carbon stabilization. The results suggest that the content of fresh organic matter that is readily utilizable by microorganisms is extremely low and the composition of DOM shifts toward a later stage dominated by relatively stable humic substances. This transition toward more stable, “aged” intermediate products reflects the preservation and carbon sequestration potential of the soil DOM system. Protein-like substances, characterized by simple structures and high bioavailability, are easily decomposed and utilized by microorganisms. After microbial degradation, these protein-like substances in organic materials and DOM are transformed into humic substances with stronger polymerization and higher stability [44]. Lei et al. [45] conducted an organic matter decomposition experiment in the black soil region of Northeast China, demonstrating that the relative content of protein-like components in DOM significantly decreased after 2 years of maize and soybean straw decomposition, whereas the relative content of humic-like components increased. This result reveals the fundamental transformation pattern of the DOM from active to stable components following the straw return to the field.

### 4.3. Influence of Different Treatments on Fluorescence Spectral Characteristics

The fluorescence indexes further indicated that the average humification index also increased more in the soybean growing season (0.87) than in the maize growing season (0.70), indicating a higher degree of humification, although both remained at relatively low levels. The fluorescence indexes further showed that WSOC was influenced by both allochthonous and autochthonous sources, with a stronger contribution from externally added straw, whereas the overall humification remained weak. The fluorescence index values were lower in the maize growing season than in the soybean growing season, reflecting the combined influence of autochthonous and allochthonous organic matter, with a stronger contribution from terrestrial sources derived from externally added materials. The lower BIX suggested strong autochthonous characteristics in the maize season and weak autochthonous characteristics in the soybean growing season. However, straw addition to Albi-Boric argosols has shown that deeper straw incorporation is associated with a lower degree of soil humification [46], which is likely due to the difference in soil types and textures, influencing decomposition dynamics. In this study, the humification coefficient increased with the soil depth, suggesting the accumulation of more stable metabolic by-products within the soil profile. During organic matter decomposition, readily available compounds, such as soluble sugars, amino acids, and other active substances, are rapidly released, whereas difficult-to-decompose substances, such as lignin and cellulose, decompose slowly at later stages. This process leads to increased humification and reduced bioavailability [47].

## 5. Conclusions

Under the maize–soybean rotation system examined in this experiment, both the SS and DS treatments during the maize residue incorporation season increased the SOC and WSOC in the 0–40 cm soil layers, while also elevating the proportion of WSOC within the total organic carbon. In the maize growing season, straw incorporation resulted in a higher abundance of highly active, protein-like components. By the subsequent soybean growing season, these components were gradually transformed into more complex and stable carbon structures, thereby contributing to the formation of a stable carbon pool in the soil. In summary, the maize–soybean rotation system mitigates challenges associated with continuous maize straw incorporation, such as slow decomposition and impaired seedling emergence, while also promoting the balanced utilization of soil nutrients, controlling pests and diseases, and enhancing carbon pool stability. Consequently, this system offers significant economic and ecological benefits.

## Figures and Tables

**Table 1:** The mean fluorescence spectral indices of soil WSOC in different treatments.

			FI	BIX	HIX
Maizeseason	SM	0–20 cm	1.53 ± 0.01 a	1.79 ± 0.40 a	0.51 ± 0.08 b
	SS	0–20 cm	1.53 ± 0.03 a	1.15 ± 0.38 b	0.70 ± 0.12 a
	DS	0–20 cm	1.53 ± 0.01 a	0.84 ± 0.12 b	0.79 ± 0.07 a
	SM	20–40 cm	1.53 ± 0.01 a	1.11 ± 0.05 a	0.70 ± 0.02 b
	SS	20–40 cm	1.57 ± 0.05 a	0.92 ± 0.14 a	0.75 ± 0.05 ab
	DS	20–40 cm	1.56 ± 0.01 a	0.90 ± 0.09 a	0.78 ± 0.04 a
Soybeanseason	SM	0–20 cm	1.64 ± 0.06 a	0.74 ± 0.11 a	0.86 ± 0.06 a
	SS	0–20 cm	1.53 ± 0.01 b	0.61 ± 0.03 a	0.89 ± 0.01 a
	DS	0–20 cm	1.57 ± 0.03 ab	0.72 ± 0.10 a	0.84 ± 0.03 a
	SM	20–40 cm	1.62 ± 0.03 a	0.64 ± 0.01 b	0.91 ± 0.01 a
	SS	20–40 cm	1.56 ± 0.04 a	0.63 ± 0.02 b	0.86 ± 0.03 b
	DS	20–40 cm	1.57 ± 0.03 a	0.69 ± 0.03 a	0.87 ± 0.01 b

Note: The values were mean ± standard deviation, *n* = 3. Different lowercases represent a significant difference on different treatments of the same soil layer (*p* < 0.05). FI: The fluorescence index, BIX: the biological index, HIX: humification index; SM: straw mulching; SS: shallow straw incorporation; and DS: deep straw incorporation.

## Data Availability

The raw data supporting the conclusions of this article will be made available by the authors on request.
